# Adiponectin is associated with cardiovascular disease in male renal transplant recipients: baseline results from the LANDMARK 2 study

**DOI:** 10.1186/1471-2369-10-29

**Published:** 2009-10-12

**Authors:** Mohd O Kaisar, Kirsty Armstrong, Carmel Hawley, Scott Campbell, David Mudge, David W Johnson, John B Prins, Nicole M Isbel

**Affiliations:** 1 Department of Nephrology Princess Alexandra Hospital, Brisbane, Queensland, Australia; 2 Diamantina Institute for Cancer, Immunology and Metabolic Medicine University of Queensland, Princess Alexandra Hospital, Brisbane, Queensland, Australia; 3 Centre of Clinical Research and Excellence, University of Queensland, Brisbane, Australia

## Abstract

**Background:**

Adiponectin is a major adipocyte-derived protein with insulin-sensitizing, anti-inflammatory and anti-atherogenic properties. Adiponectin levels correlate inversely with renal function and higher levels are predictive of lower cardiovascular disease (CVD) in patients with normal renal function and chronic kidney disease. No data exists on the association between adiponectin and CVD in renal transplant recipients (RTR).

**Methods:**

Standard biochemistry, clinical data and adiponectin were collected from 137 RTR recruited to the LANDMARK 2 study at baseline. The LANDMARK 2 study is an ongoing randomized controlled study that compares the outcome of aggressive risk factor modification for cardiovascular disease versus standard post-transplant care in renal transplant recipients with impaired glucose tolerance or diabetes mellitus.

**Results:**

Mean patient age was 53.4 ± 12 years and the median post-transplantation period was 5 (0.5-31.9) years. Mean serum adiponectin level was 12.3 ± 7.1 μg/mL. On univariate analysis, adiponectin was positively associated with female gender (P = 0.01) and serum high-density lipoprotein (HDL) concentration (P < 0.001), and inversely with body mass index (P = 0.009), metabolic syndrome (P = 0.047), abnormal glucose tolerance (P = 0.01), C-reactive protein (P = 0.001) and serum triglyceride (P < 0.001). On stepwise multivariate analysis, adiponectin in males was negatively correlated with combined baseline CVD (P = 0.03), waist-hip ratio (P = 0.003) and glomerular filtration rate (P = 0.046), and positively with HDL (P < 0.001). In contrast, in females adiponectin was inversely associated with C-reactive protein (P = 0.001) and serum triglyceride.

**Conclusion:**

In conclusion, adiponectin is positively correlated with inflammation, dyslipidemia and abnormal glucose tolerance in RTR. Furthermore, hypoadiponectinemia correlated with increased baseline CVD in male RTR.

## Background

Cardiovascular disease (CVD) remains a major cause of premature death in patients with chronic kidney disease (CKD), including renal transplant recipients (RTR). RTR in the age group of 35 to 44 year olds have up to a 10-fold increase in cardiovascular death with nearly double that rate within the ages of 55 to 64 [1]. Adiponectin is an abundant multifunctional adipocyte derived protein with anti-inflammatory, anti-atherogenic and insulin sensitizing activity [2]. Adiponectin has sequence homology to the C1q family of complement proteins and circulates in low, medium and high molecular weight forms. Clinical data indicates that the high molecular weight form strongly correlates with improved insulin sensitivity and glucose tolerance than total adiponectin levels [3]. Adiponectin levels have been shown to be lower in males, obesity, insulin resistance, in patients with type 2 diabetes mellitus, coronary artery disease and essential hypertension [4-7]. In contrast, adiponectin is elevated in kidney disease including nephrotic syndrome [8], and particularly in end-stage kidney disease, with levels up to three times higher compared to the normal population [9]. The rise in adiponectin with declining glomerular filtration rate (GFR) is hypothesized to be secondary to diminished renal elimination, though the exact role of the kidney in the biodegradation and excretion of adiponectin remains unclear. Recent observational studies have shown adiponectin to be a novel risk marker of CVD in patients with stage 1 to 5 CKD. These prospective studies have revealed that low adiponectin levels to be predictive of the development of new cardiovascular events in patients with CKD, including the hemodialysis population [9-11] While adiponectin has been shown to predict the development of type 2 diabetes in renal transplant recipients [12], there is yet to date any published data on the association between adiponectin and CVD in RTR. We examined the relationship between cardiovascular disease and adiponectin in RTR recruited to participate in the LANDMARK 2 study.

## Materials and methods

### Study Design and Population

The Longitudinal Assessment of Numerous Discrete Modifications of Atherosclerotic Risk Factors in Kidney disease 2 (LANDMARK 2) study is an ongoing 2 year, open label, single centre randomized controlled trial. Conducted at the Princess Alexandra Hospital in Brisbane, Australia it compares aggressive risk factor modification for cardiovascular disease versus standard post-transplant care in renal transplant recipients with impaired glucose tolerance or diabetes mellitus. Targets include aggressive cholesterol and homocysteine reduction, blood pressure, diabetic control and weight loss. Comparison will be made to a control group with normal glucose tolerance who will receive standard post-transplant care, as determined by a normal oral glucose tolerance test. Therefore, our analysis included 102 patients with abnormal glucose tolerance from the LANDMARK 2 study population and 35 matched RTR with normal glucose tolerance from the substudy group which served as the control arm. All eligible patients provided informed consent to participate in the study, which was approved by the Human Research Ethics Committee of the University of Queensland and Princess Alexandra Hospital. Impaired glucose tolerance or diabetes was defined according to the World Health Organization specifications [13].

### Clinical Data

Demographic data were recorded, including age, sex, race, cause of renal disease, duration of current transplant and baseline cardiovascular disease. Assessment of cardiovascular risk factors included any of the following before or after transplantation: (1) Ischemic heart disease (acute coronary syndrome requiring hospitalization, non-fatal myocardial infarction, percutaneous coronary intervention or coronary artery bypass graft surgery), (2) peripheral vascular disease (angioplasty, bypass or amputation), (3) cerebrovascular event (transient ischemic attack or stroke with neurologic deficit), (4) hypertension (previous or current use of antihypertensive agents or self reported), (5) diabetes (previous or current use of oral hypoglycemic agents or insulin) and (6) dyslipidemia (previous or current use of lipid-lowering therapy or self reported). Metabolic syndrome (MS) was defined according to the National Cholesterol Education Panel Adult Treatment Panel III (NCEP) criteria [14].

### Clinical Measurements

Blood pressure (BP), height, weight, waist and hip circumference were measured using standardized equipment. Body mass index (BMI) and waist-hip ratio (WHR) were determined. Central obesity was defined as waist circumference ≥ 102 cm in men or 88 cm in women [15].

### Biochemical Analysis

Blood specimens were collected after an 8-hour overnight fast. Serum concentrations of creatinine, hemoglobin, glucose, insulin, HbA1c, hsC-reactive protein, homocysteine, adiponectin and lipids [total cholesterol, low density lipoprotein (LDL), high density lipoprotein (HDL), very low density lipoprotein (VLDL), Apolipoprotein a, Apolipoprotein b and triglycerides] were measured using standard techniques. Homeostasis Model Assessment of insulin resistance (HOMA) was calculated using the standard formula = [Fasting insulin (μU/mL) × fasting blood glucose (mmol/L) ÷ 22.5]. Serum adiponectin was measures using the LINCO method (radioimmunoassay technique-lower limit of detection 1 ng/mL; coefficient of variance 6.6%).

### Statistical Analysis

Statistical analysis was performed using standard statistical software (Stata 10 SE, StataCorp LP 2007). Results are expressed in mean ± SD, median (interquartile range [IQR]), or frequencies (%), depending on data distribution. *A priori *P < 0.05 was considered to be statistically significant. Log transformation was performed for non-parametric predictor variables. Univariate and multivariate analysis was performed to determine which demographic, clinical and biochemical parameters were associated with serum adiponectin. Variables with *P *< 0.1 on univariate analysis, which were not collinearly related, were then entered into a backward elimination procedure utilizing likelihood ratio until the most parsimonious model was identified. Multivariate modeling was performed stratified for gender because of published data demonstrating associations between adiponectin to be strongly gender dependent. Regression diagnostics, including plots of residuals against the explanatory variables and against the fitted values, were carried out and tests for normal distribution of the residuals were performed. Variance inflation factor was checked as an index of collinearity.

## Results

Table 1 shows the baseline demographic characteristics of all subjects from the LANDMARK 2 study. The mean patient age was 53.4 years, with a larger proportion of male participants and the median duration of kidney transplantation was 5 (0.5 to 31.8) years. Fifty six (41.7%) participants had type 2 diabetes while 46 (33.6%) patients had impaired glucose tolerance. For the purpose of analysis, the two groups were combined as the abnormal glucose tolerance group. Most subjects (91%) had a history of hypertension which was well controlled, with a mean blood pressure of 133/77 mmHg. The mean GFR was 51.8 mls/min/1.73 m^2 ^and the majority of RTR had stage 2 (57.7%) and 3 (29.2%) CKD. Serum adiponectin was missing for 3 subjects. The mean adiponectin concentration was 12.3 ± 7.1 μg/mL. After excluding subjects with polycystic kidney disease, due to the unpredictability in obtaining an accurate measurement of waist circumference, 59 (43.1%) subjects had metabolic syndrome as defined by the NCEP criteria. Combined cardiovascular disease (acute coronary syndrome, myocardial infarction, coronary artery bypass graft surgery, stroke and peripheral vascular disease) was present at baseline in 35 subjects (26%).

**Table 1:** Baseline characteristics of 137 subjects in the LANDMARK 2 study

Characteristics	Value
Age (yrs)	53.4 ± 11.1
Sex M/F, n	85/52
Body mass index (kg/m^2^)	28.1 ± 5.4
Abnormal glucose tolerance, n (%)	102 (75)
Duration of transplant (yrs)	7.8 (0.5-31.9)
Hypertension, n (%)	125 (91)
Systolic blood pressure (mmHg)	132.6 ± 15.2
Diastolic blood pressure (mmHg)	77.7 ± 10.5
CKD stage, n (%)	
1 (>90 mls/min)	13 (9.5)
2 (60-90 mls/min)	79 (57.7)
3 (30-60 mls/min)	40 (29.2)
4 (15-30 mls/min)	5 (3.7)
MDRD Glomerular filtration rate (mls/min/1.73 m^2^)	51.8 ± 18.3
Fasting glucose (mmol/L)	
-Abnormal glucose tolerance group	6.1 ± 2.2
-Normal glucose tolerance group	4.9 ± 4.5
Fasting cholesterol (mmol/L)	4.9 ± 1.1
Fasting triglyceride (mmol/L)	1.9 ± .9
C-reactive protein (mg/L)	8.0 ± 12.6
Adiponectin (μg/mL)	12.3 ± 7.1
Metabolic syndrome, n (%)	59 (43.1)

Combined baseline cardiovascular events (ACS, MI, CABG, stroke, PVD), n (%)	35 (26)

* Plus-minus values are mean ± SD. CKD-Chronic kidney disease; MDRD-Modification of Diet in Renal Disease; GFR-glomerular filtration rate; ACS-acute coronary syndrome; MI-myocardial infarction; CABG-coronary artery bypass graft surgery; PVD-peripheral vascular disease

C-reactive protein (CRP) and random urinary protein creatinine ratio were skewed and hence presented in tertiles. On univariate analysis adiponectin levels were positively correlated with female gender, HDL and Apolipoprotein a (Table 2). Adiponectin levels correlated inversely with BMI, WHR, abnormal glucose tolerance, metabolic syndrome, serum triglyceride, VLDL and CRP. There was no significant correlation between adiponectin, fasting insulin (P = 0.6) and HOMA scores (P = 0.9), although these data were only available for 81 patients (59%). Furthermore, we failed to show any significant relationship between serum adiponectin and measures of kidney function, including serum creatinine, CKD stage and GFR on univariate analysis. In addition, we were unable to highlight any significant association with the use of medications known to influence serum adiponectin levels, including prednisolone, angiotensin converting enzyme (ACE) inhibitors and angiotensin receptor blockers (ARB). The majority of our patients received calcineurin inhibitors (CNIs) as the primary immunosuppression therapy. There was no significant association between either a class effect of CNIs or individual treatment with either cyclosporin or tacrolimus on serum adiponectin levels.

**Table 2:** Variables associated with adiponectin on univariate linear regression analysis in the LANDMARK 2 population

	Univariate Analysis		
Variable	*β*	P	95% CI
Sex	3.24	0.01	7.71-5.71
Body mass index (kg/m2)	-0.30	0.009	-0.53 to -0.08
Abnormal glucose tolerance	-3.67	0.008	0.95-6.40
Metabolic syndrome	-2.47	0.047	-4.91 to -0.03
Combined cardiovascular events	-2.03	0.16	-4.84 to 0.79
Waist hip ratio (cm)	-21.48	0.001	-33.06 to -9.90
Log fasting glucose (mmol/L)	-4.55	0.04	-8.60 to -0.49
Log triglyceride (mmol/L)	-6.10	<0.001	-8.63 to -3.56
Log HDL (mmol/L)	11.05	<0.001	7.23 to 14.88
VLDL (mmol/L)	-5.99	0.001	-9.48 to -2.49
Apolipoprotein a (g/L)	10.93	<0.001	5.63 to 16.23
C-reactive protein (mg/L)			
0.9-2	*ref*	*ref*	*ref*
2.1-4.7	-1.15	0.44	-4.07 to 1.77
4.8-84	-4.03	0.007	-6.95 to -1.11
Urinary PCR (mg/L)			
5-14	*ref*	*ref*	*ref*
15-35	0.18	0.91	-3.10 to 3.47
36-1000	-2.27	0.15	-5.40 to 0.85
GFR (mls/min/1.73 m2)	-0.45	0.18	-0.11 to 0.02
ACE inhibitor (mg/day)	-2.12	0.1	-4.70 to 0.45
ARB (mg/day)	2.70	0.1	-0.51 to 5.90

*β-coefficient; Protein creatinine ratio (PCR)

Results of multivariate linear regression analysis, stratified for gender are outlined in Table 3. In male RTR, adiponectin levels were positively associated with HDL (*β *= 8.56, P = 0.003). In contrast, the relationship between adiponectin and both waist hip ratio (*β *= -18.3, P = 0.03) and GFR were inverse, with the strongest correlation observed for the relationship between adiponectin and WHR in the regression model. There was a non-significant trend towards an association between adiponectin and CRP in male subjects. A major finding in our analysis was the significant correlation between adiponectin and baseline combined CVD in male RTR (*β *= -3.38, P = 0.03) with stepwise multivariate linear regression analysis. In female RTR, there was a highly significant negative relationship between adiponectin, serum triglyceride and CRP.

**Table 3:** Variables associated with adiponectin on multivariate linear regression analysis in the LANDMARK 2 population

Variable	Male			Female		
	*β*	P	95%CI	*β*	P	95% CI
Log HDL (mmol/L)	8.56	0.003	3.01-14.12			
Log triglyceride (mmol/L)				-6.29	0.007	-10.74 to -1.84
GFR (mls/min/1.73 m^2^)	-0.07	0.046	-0.14 to -0.001			
CRP (mg/L)						
Tertile 1	*ref*	*ref*	*ref*	*ref*	*ref*	
Tertile 2	-2.60	0.1	-5.70 to 0.51			
Tertile 3	-3.26	0.07	-6.74 to 0.21	-6.56	0.007	-10.70 to -2.43
						
Waist hip ratio	-18.30	0.003	-34.68 to -1.89			

Combined CVD	-3.38	0.03	-6.48 to -0.28			

*ref = reference; β-coefficient

## Discussion

This study to our knowledge is the first to demonstrate a significant link between lower adiponectin levels and CVD in the renal transplant population. We demonstrated that in male RTR with predominantly stage 2 and 3 CKD there is a strong independent *inverse *association between serum adiponectin levels and an increased prevalence of cardiovascular disease. Our study also showed an inverse correlation between waist-hip ratio and GFR with serum adiponectin in males whilst in females adiponectin was inversely associated with CRP and serum triglycerides.

Our findings related to CVD risk parallels similar results from other studies in both the non-transplant population with normal kidney function and CKD. Adiponectin levels have been shown to vary inversely with GFR and CVD, suggesting a potential protective role for adiponectin against the development of cardiovascular events in patients with kidney disease. This association has been highlighted in several recent studies. Zoccali et al showed that in their cohort of 227 hemodialysis patients, that each 1 μg/mL increase in adiponectin concentration was associated with a 3% risk reduction in new cardiovascular events [9]. Analogous results have been observed in a study by Becker et al, from the Mild to Moderate Kidney study database in non-diabetic subjects with primarily stage 3 to 4 CKD [10], where patients who developed new cardiovascular events had significantly lower adiponectin levels (odds ratio, 0.72; 95% CI 0.58 to 0.89; P = 0.002). In a recent study, Iwashima et al showed that adiponectin levels were significantly higher in patients with more severe kidney impairment as assessed by CKD stage [11]. After dividing the patients into a high and low group based upon gender-specific median values of adiponectin, the low adiponectin group had a significantly shorter event-free survival compared to the higher group (<4.39 μg/ml in men, <6.84 μg/ml in women, P < 0.03). In contrast to the above findings, Menon et al, in their analysis of 820 patients with a GFR range of between 13 and 55 mls/min from the MDRD (Modified Renal Diet) database revealed a direct correlation between adiponectin and the relative risk of cardiovascular mortality [16]. In multivariable adjusted Cox models, a 1 μg/ml increase in adiponectin was associated with a 3% (hazard ratio 1.03; 95% CI 1.01 to 1.05; P = 0.02) elevated risk for all-cause and 6% (hazard ratio 1.06; 95% CI 1.03 to 1.09; P = 0.001) higher risk for cardiovascular mortality. The direct correlation between adiponectin and CVD in the MDRD study may be due to a residual confounding effect of reduced renal function or processes accompanying CKD, but unadjusted for reduced GFR. For example, the mean GFR and adiponectin in the MDRD was 32 mls/min and 6 μmol/L respectively, in comparison to 63 mls/min and 12 μmol/L correspondingly in the study by Becker et al, highlighting the strong association between adiponectin and GFR [17].

Adiponectin has been shown to possess anti-inflammatory and metabolic effects. Advantageous metabolic properties include improvement in glucose tolerance, higher levels of HDL and reduction in triacylglycerol rich lipoproteins, a hallmark feature of obesity related dyslipidemia [18-20]. In our study, on multivariate analysis we showed a positive association between adiponectin and HDL in male RTR while the converse relationship with TG was restricted to female subjects. We also demonstrated that adiponectin varied inversely with CRP in our female cohort of RTR (P = 0.007), whilst there was a non-statitistically similar trend between CRP and adiponectin in our male patients (P = 0.07). In multiple studies, adiponectin has been shown to be inversely associated with markers of inflammation including CRP [21-23]. Higher adiponectin levels in CKD may represent a compensatory process to the inflammatory milieu in the kidney disease. Adiponectin has also been shown to exhibit anti-inflammatory activity in atherosclerotic experimental models [24,25]. In addition, adiponectin ameliorates VSMC proliferation by inhibition of mitogen-activated protein kinase, increases production of nitric oxide and tissue inhibitor of metalloproteinase-1, resulting in vasodilatation and decrease in plaque rupture respectively [26-28].

Our study has several limitations. Firstly, as the findings from this study are derived from observational data, a cause and effect inference cannot be made between the study variables. Furthermore, while our study size was small, it is similar in number to previously cited studies in CKD patients that assessed the association between adiponectin and CVD and with similar outcome findings. In addition, the smaller number of female participants may obscure a potentially significant interaction between adiponectin and CVD in this group, though we believe the likelihood of a type 2 error is low and the results are statistically and biologically genuine. In addition, the HMW isoform was not measured as current assays for the HMW form are not standardized and unreliable.

The strengths of our study include the statistical adjustment performed for other factors associated with an increased risk of CVD such as abnormal glucose tolerance, dyslipidemia, hypertension and inflammation. We also performed stratified regression analyses according to gender based upon previous published studies that confirmed an association between adiponectin and cardiovascular events in male subjects, a higher incidence of CVD in men compared to women and a significant difference in adiponectin between the genders on univariate analysis.

Our findings suggest that higher adiponectin levels may be protective against the development of CVD in male renal transplant recipients. The observed relationship between hypoadiponectinemia and increased CVD in male RTR may be explained by several factors. As adiponectin is positively correlated with HDL, low HDL levels in hypoadiponectenemia may partly account for the increased risk of CVD. The smaller number of female patients may have obscured a similar finding in this group. There exists a biological rationale for increasing adiponectin levels in an attempt to improve outcomes from atherosclerotic disease in this population. Several medications, including ACE inhibitors, ARB and thiazolidinediones have been shown to increase adiponectin levels in patients with normal kidney function and CKD [29-31]. However, there have been no published studies on the effect of increasing adiponectin levels and subsequent cardiovascular outcomes. In conclusion, it is important that future studies continue to delineate the biological relevance of adiponectin either as a biomarker of vascular disease or potential therapeutic agent in improving cardiovascular outcomes in RTR and other populations with high cardiovascular risk.

## Competing interests

The authors declare that they have no competing interests.

## Authors' contributions

MK Statistical analysis of paper, first author, reviewed subjects in clinic and collection of clinical data.

KA Assisted in design of study, analysis of data, reviewed subjects in clinic and collection of clinical data.

HC Statistical analysis, drafting and revision of paper for publication.

SC Assisted in drafting of manuscript and revision of paper for publication.

DM Assisted in drafting of manuscript and revision of paper for publication.

DJ Co-investigator, design of study, drafting and revision of paper for publication.

JP Co-investigator, design of study, drafting and revision of paper for publication.

NI Primary investigator, design of study, drafting and revision of paper for publication.

**All authors read and approved the final manuscript**.

## Pre-publication history

The pre-publication history for this paper can be accessed here:

http://www.biomedcentral.com/1471-2369/10/29/prepub
